# Complete agenesis of the infra‐renal aorta

**DOI:** 10.1002/ccr3.4988

**Published:** 2021-12-26

**Authors:** Charalampos Seretis, Chrysanthi P. Papageorgopoulou, Konstantinos M. Nikolakopoulos, Andreas L. Tsimpoukis, Spyros I. Papadoulas

**Affiliations:** ^1^ Department of Vascular Surgery University General Hospital of Patras Patras Greece

**Keywords:** agenesis, aorta, surgery, vascular

## Abstract

Complete agenesis of the infra‐renal aorta is an extremely rare anatomical variant, and its presence can complicate the accurate interpretation of the aortic sonographic assessment.

## Graphical abstract

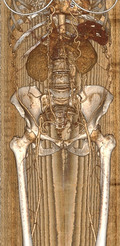

## 1 CASE DESCRIPTION

Agenesis of the infra‐renal aorta is very uncommon anatomical variation; the presence of which can complicate significantly the accurate interpretation of the sonographic assessment of the aorta, leading to false initial differential diagnoses such as extensive aortic thrombosis or even contained rupture.

Complete agenesis of the infra‐renal aorta is an extremely rare anatomical variation, with only few cases reported in the international literature to date.^1^, ^2^ Herein, we present a case in which this rare condition was sonographically diagnosed as possible complete aortic thrombosis or contained rupture, highlighting the need for considering this anatomical variation as a potential explanation in similar cases of unconventional initial sonographic findings of the infra‐renal aortic segment.

**FIGURE 1:**
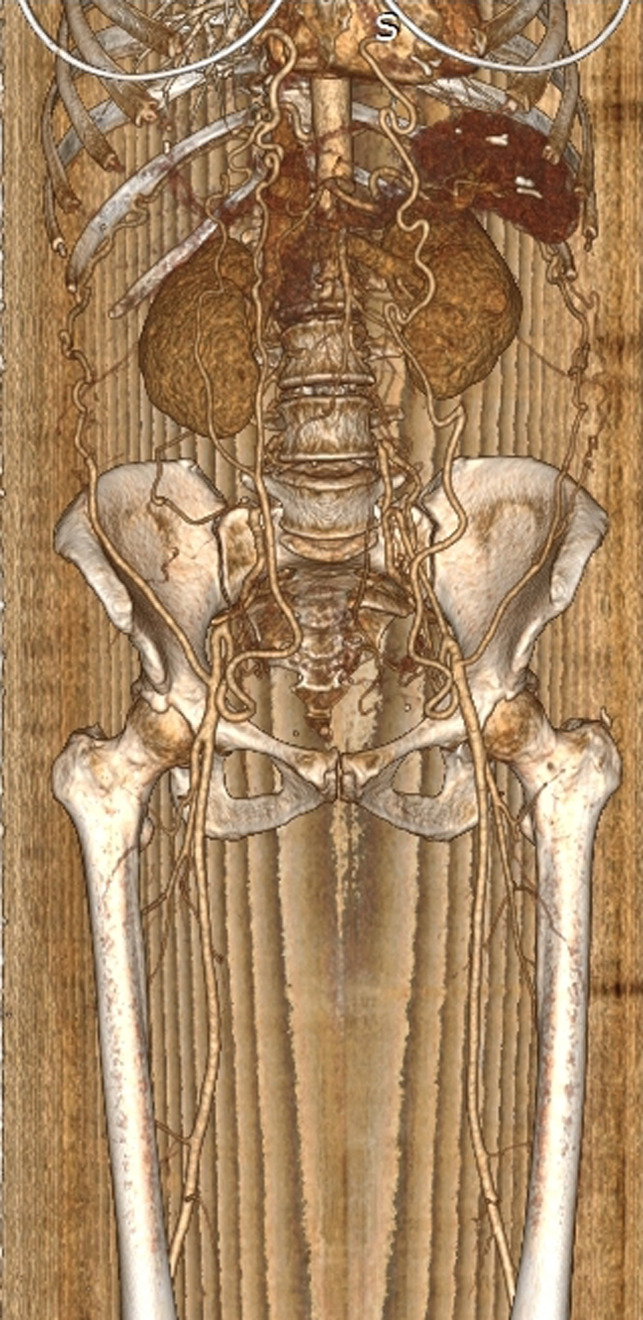
3D reconstruction of the CT angiogram, demonstrating complete absence of the infra‐renal aortic trunk, which is substituted by a pair of enlarged lumbar arteries (red arrows) supplying the internal iliac arteries' territory; please note the ectatic bilateral inferior epigastric arteries compensating for the undeveloped aorto‐iliac axes

A 48‐year‐old Caucasian female patient presented to our emergency department with features of new‐onset, mild bilateral calf edema. Her past medical history was unremarkable. A vascular consult was sought to rule out the presence of ileo‐femoral or more proximal venous thrombosis as part of the initial diagnostic work‐up. Her admission blood tests (full blood count, renal and liver function tests, and coagulation profile) were within normal range; bedside lower limb venous triplex did not demonstrate any signs compatible with proximal venous thrombosis; however—despite multiple attempts—the infra‐renal part of the aorta could not be visualized till the level of common femoral vessels. As the latter raised the possibilities of extensive thrombosis or even contained rupture of the aorta, an urgent computed tomography angiogram (CTA) was performed. The later demonstrated complete agenesis of the aortic trunk distally to the level of the renal arteries, with substitution of the undeveloped part of the aorta by a pair of significantly enlarged lumbar arteries, along with significant prominence of the superior‐inferior epigastric anastomotic circuit bilaterally, as an adjustment to the absence of developed aorto‐iliac axes (Figure 1). The patient was reassured from the vascular point of view and a note was sent to her general practitioner, highlighting the need for clear documentation of this rare anatomical variation in the patient's medical records; particularly, should she require any vascular intervention in her future life. Of note, the patient remains completely asymptomatic at 30 days’ follow‐up.

## CONFLICTS OF INTEREST

The authors declared no potential conflicts of interest with respect to the research, authorship, and/or publication of this article.

## AUTHOR CONTRIBUTIONS

CS, CP, and AT contributed to the clinical data collection and prepared the case report; KN and SP contributed to the design of the case report presentation and performed the final revision of the manuscript. All listed co‐authors approve the manuscript in its final form.

## CONSENT

Informed consent was obtained from the patient and is available upon request by the editorial office; no ethical committee approval was required for the publication of this case report.

## Data Availability

The authors declare that the supporting data for this case are presented within the manuscript.
